# Hospital-Based Surveillance of Respiratory Pathogen Positivity and Distribution: A Six-Year Epidemiological Study in Beijing, 2020–2025

**DOI:** 10.3390/jcm15218742

**Published:** 2026-11-09

**Authors:** Nan Duan, Jialin Du, Le Wang, Xujun Liu, Haiming Huang, Haixia Li, Xi Yao, Bo Jin

**Affiliations:** 1 Department of Clinical Laboratory, Peking University First Hospital, Beijing 100034, China; 2 Department of Infection Prevention and Control, Peking University First Hospital, Beijing 100034, China

**Keywords:** respiratory tract infections, respiratory pathogens, positivity, epidemiology

## Abstract

**Objective**: Respiratory tract infections (RTIs) are imposing a great economic burden, morbidity, and mortality on the healthcare system. Eleven common respiratory pathogens, based on age of onset, clinical setting, season and calendar year during and after the COVID-19 pandemic, were analyzed to optimize clinical diagnosis and prevention. **Methods**: In this retrospective cross-sectional study, respiratory pathogen testing results of 9882 samples from RTI patients from January 2020 to December 2025 were included to statistically analyze the positivity, distribution and trend of change. Multiplex PCR combined with capillary electrophoresis was used to simultaneously detect 11 respiratory targets. **Results**: The overall positive detection rate was 24.71%, with the top three pathogens being human rhinovirus (HRV) (6.20%), influenza A virus (InfA) (4.28%), and respiratory syncytial virus (RSV) (4.13%). The patients aged under 18 had the highest detection rates (≤6 years: 39.80%; 7 to 17 years: 37.95%). HRV, RSV and *Mycoplasma pneumoniae* (MP) were the primary pathogens among pediatric patients, while InfA was predominant in adults (including those >65 years). The 11 pathogens were most common in emergency department patients (29.14%), followed by outpatient patients (28.57%). InfA was the most predominant virus, followed by HRV and RSV, in the intensive care unit, emergency department, and outpatient settings. The detection rate was highest in autumn (33.22%), followed by winter (26.61%). MP had the highest rate in autumn, and InfA was the most frequent in winter. During the COVID-19 pandemic (2020–2022), the respiratory pathogen spectrum was dominated by HRV, RSV, and human parainfluenza virus (HPIV). And the prevalent pathogens shifted to InfA, MP and HRV during 2023–2025. Co-detections were observed in 8.03% of the positive cases, and 82.65% occurred in patients aged < 18 years. The dominant co-detections were HRV + MP, HRV + RSV and HRV + HPIV. **Conclusions**: The distribution of respiratory pathogens exhibited specific variations based on age, clinical setting, season, and calendar year, suggesting that differentiated prevention should be developed for different patients.

## 1. Background

Respiratory tract infections (RTIs) remain a predominant cause of global morbidity and mortality, particularly among the elderly and young children, and they encompass a tremendous breadth of clinical disease [1]. Although many respiratory infections are mild and self-limiting, it is indicated that lower respiratory tract infections (LRTIs) became the only communicable disease among the top 10 causes of death, while the other nine were noncommunicable diseases (NCDs) in upper-middle-income and high-income countries [2]. Worldwide, infectious diseases, including acute respiratory infections, continue to be the leading causes of death, especially for children under the age of five [3]. In 2023, there were 2.50 million deaths and 98.7 million disability-adjusted life-years (DALYs) caused by LRTIs [4]. In China, RTIs have remained a threat, and it is estimated that there were 206,930.22 deaths from non-COVID-19 LRTIs, with an all-age mortality rate of 14.54 deaths per 100,000 population in 2021 [5]. Apart from population-based respiratory infection prevention measures, morbidity and mortality could be reduced by early identification of infected patients at risk of developing severe disease [1].

Appropriate management of RTIs usually depends on accurately identifying the offending pathogens as viral, bacterial, fungal, or other classes. However, sometimes viruses and bacteria exhibit almost indistinguishable symptoms or overlapping clinical features in respiratory infections [6]. In addition, viral infections can induce host immune responses, which promote bacterial proliferation, complicating clinical diagnosis [7], and a lack of accurate and timely diagnosis might result in the overuse of antimicrobial agents. The mechanisms of post-viral bacterial infection are complex, and they are thought to be associated with direct mucosal/epithelial damage by respiratory viral infections, increased bacterial colonization of the upper and lower respiratory tracts, and dysregulation of immune responses, which might comprise multifactorial processes mediated by interactions between viruses, bacteria, and the host immune system [7]. Therefore, conducting epidemiological investigations on respiratory pathogens is vital for accurate diagnosis and proper treatment.

The effective management of RTIs depends on the timely identification of pathogens through respiratory specimens, a procedure that employs traditional culture-based means to detect the offending microorganisms. For common respiratory tract viruses and bacteria that are difficult to culture, antigen-based testing and viral isolation cultures are not sensitive or rapid diagnostic methods. Molecular detection methods not only shorten the turnaround time but also allow for the simultaneous detection of multiple pathogens, and they have become the recommended methods for diagnosing RTIs [8,9]. For instance, capillary electrophoresis-based multiplex PCR can provide an accurate and rapid method for the high-throughput detection of pathogens, allowing the identification of various viruses and bacteria associated with RTIs [10].

Currently, effective vaccines or medications have yet to be developed and approved for many respiratory tract pathogens [11,12]. The prevalence of respiratory microorganisms varies with age, geographical region, seasonal timing, climate, human mobility and viral strain, all of which can easily influence epidemiological patterns [13,14]. During the coronavirus disease 2019 (COVID-19) pandemic, the Chinese government implemented a massive roll-out of non-pharmaceutical interventions (NPIs) involving mask mandates, routine nucleic acid testing, health-code surveillance, and localized lockdowns from January 2020 through December 2022 that were effective in curbing disease transmission [15]. As the transmission of SARS-CoV-2 was reduced, other respiratory viruses (such as influenza) that spread via the same transmission route were also affected; the number of influenza cases declined when control measures were implemented [16,17,18]. And on 8 January 2023, COVID-19 management was downgraded from Class A to Class B, with full border reopening and the removal of large-scale NPIs nationwide. A better understanding of prevalent respiratory pathogens is critical for effective prevention, control, and treatment. In light of these factors, our study aimed to describe the positivity and distribution of the 11 respiratory panel targets across age groups, clinical settings, seasons and calendar years during and after the COVID-19 pandemic, in order to provide scientific evidence for the optimization of clinical diagnostic and therapeutic strategies, and for the prevention and control of public health.

## 2. Materials and Methods

### 2.1. Subjects

We conducted a retrospective cross-sectional study at Peking University First Hospital (a large-scale tertiary general hospital) from 1 January 2020 to 31 December 2025. A total of 9882 samples from pediatric and adult patients presenting with clinical signs/manifestations indicative of RTIs, including fever (≥37.5 °C), pharyngitis, cough, and influenza-like symptoms such as sore throat, runny or stuffy nose, respiratory distress, muscle aches, chills, and fatigue, were recruited in the present study. The exclusion criteria encompassed respiratory inflammation caused by non-infectious factors, hematological system disorders or immunodeficiency diseases, and incomplete clinical and laboratory data. In our study, when the same patient underwent repeated sampling, an interval within two weeks was treated as one patient observation, and the repeated tests were consolidated by retaining the first test result. Except for gender and age, all clinical and demographic data were de-identified to protect patients’ private information. This study was reviewed and approved by the Institutional Ethics Committee of Peking University First Hospital (No. 2025-research-096), and a review in terms of patient informed consent was not needed because of the retrospective observational design, according to the decision of the Institutional Ethics Committee of Peking University First Hospital. All methods in the study were performed in accordance with the relevant guidelines and regulations.

### 2.2. Sample Collection and Detection

Nasopharyngeal or oropharyngeal samples from enrolled patients for testing pathogens were collected by trained nurses in line with a standardized protocol. The respiratory specimens were stored immediately in sterile viral transport media at 4 °C and processed within 24 h. DNA and RNA were extracted using the nucleic acid extraction and purification kit by magnetic bead method (Xi’an Tian Long Science and Technology, Xi’an, China) according to the manufacturer’s protocol. The pathogen detection was carried out with a multiplex detection kit for 11 respiratory pathogens by a capillary electrophoresis fragment analysis method based on PCR (Ningbo Health Gene Technologies Co., Ltd., Ningbo, China), including influenza A virus (InfA), influenza B virus (InfB), human parainfluenza virus (HPIV), human respiratory syncytial virus (RSV), human adenoviruses (HADV), human metapneumovirus (HMPV), human rhinovirus (HRV), human bocavirus (HBoV), human coronavirus (HCoV: OC43, HKU1, NL63 and 229E), *Chlamydia* (Ch), and *Mycoplasma pneumoniae* (MP). SARS-CoV-2 was not included in the panel, and the testing results are restricted to 11 investigated pathogens. An integrated one-step RT-PCR/PCR multiplex assay using 11 sets of specific primers was performed in a single tube with nucleic acid samples, enabling the co-amplification of genomic DNA targets (from HADV, HBoV, MP, and Ch) and cDNA targets (from InfA, InfB, HPIV, RSV, HMPV, HRV, and HCoV). The protocol involves an initial reverse-transcription step to convert viral RNA into cDNA (50 °C for 15 min), followed by high-temperature denaturation and PCR cycling. After successful extraction and amplification, capillary electrophoresis fragment analysis was performed using an Applied Biosystems 3500 Genetic Analyzer (Thermo Fisher Scientific, Foster City, CA, USA).

Each experiment included an internal control, positive control, and negative control to ensure the reliability and accuracy of the detection results. The analytical limit of detection for the following 11 pathogens are as follows: InfA, 0.098 TCID_50_/mL; InfB, 2.0 TCID_50_/mL; RSV, 0.4 TCID_50_/mL; HPIV, 0.4 TCID_50_/mL; HCoV, 0.3 TCID_50_/mL; HRV, 0.15 TCID_50_/mL; HMPV, 0.35 TCID_50_/mL; HBoV, 5000 Copies/mL; HADV, 2000 Copies/mL or 1 TCID_50_/mL; Ch, 5000 Copies/mL; and MP, 3000 Copies/mL. This assay has no cross-reactivity with the following pathogens: Cytomegalovirus, Epstein–Barr virus, *Ureaplasma urealyticum*, *Staphylococcus aureus*, *Streptococcus pyogenes*, *Staphylococcus epidermidis*, *Enterococcus faecalis*, *Pseudomonas aeruginosa*, *Klebsiella pneumoniae*, *Escherichia coli*, measles virus, mumps virus, rubella virus, and varicella-zoster virus. SARS-CoV-2 was not included in this assay, and reliably linked SARS-CoV-2 results were unavailable for the present analysis. A negative panel result indicates that none of the 11 tested targets were detected, and it does not exclude other respiratory pathogens.

### 2.3. Statistical Analysis

Descriptive and summary statistics were described and performed using R software (Version 4.3.1). A non-normally distributed continuous variable (age) was presented as median (25th–75th percentiles). The distribution of pathogens and categorical variables was presented as frequencies with percentages, and the Chi-square (χ2) tests were applied to analyze the frequency data among the study groups. *p*-values were adjusted for multiple comparisons using the false discovery rate (FDR) correction by Benjamini–Hochberg procedure, with significance defined as q < 0.05. Multivariable logistic regression was performed to identify factors associated with pathogen detection. Variables included age group, sex, clinical setting, season, and calendar year (reference categories: adults age 18–44 years, male, inpatient setting, spring, and year 2022). Adjusted odds ratios (aORs) and 95% confidence intervals (CIs) were calculated.

## 3. Results

### 3.1. Patient Characteristics and Overall Detection Percentage of the 11 Pathogens

**Figure 1:**
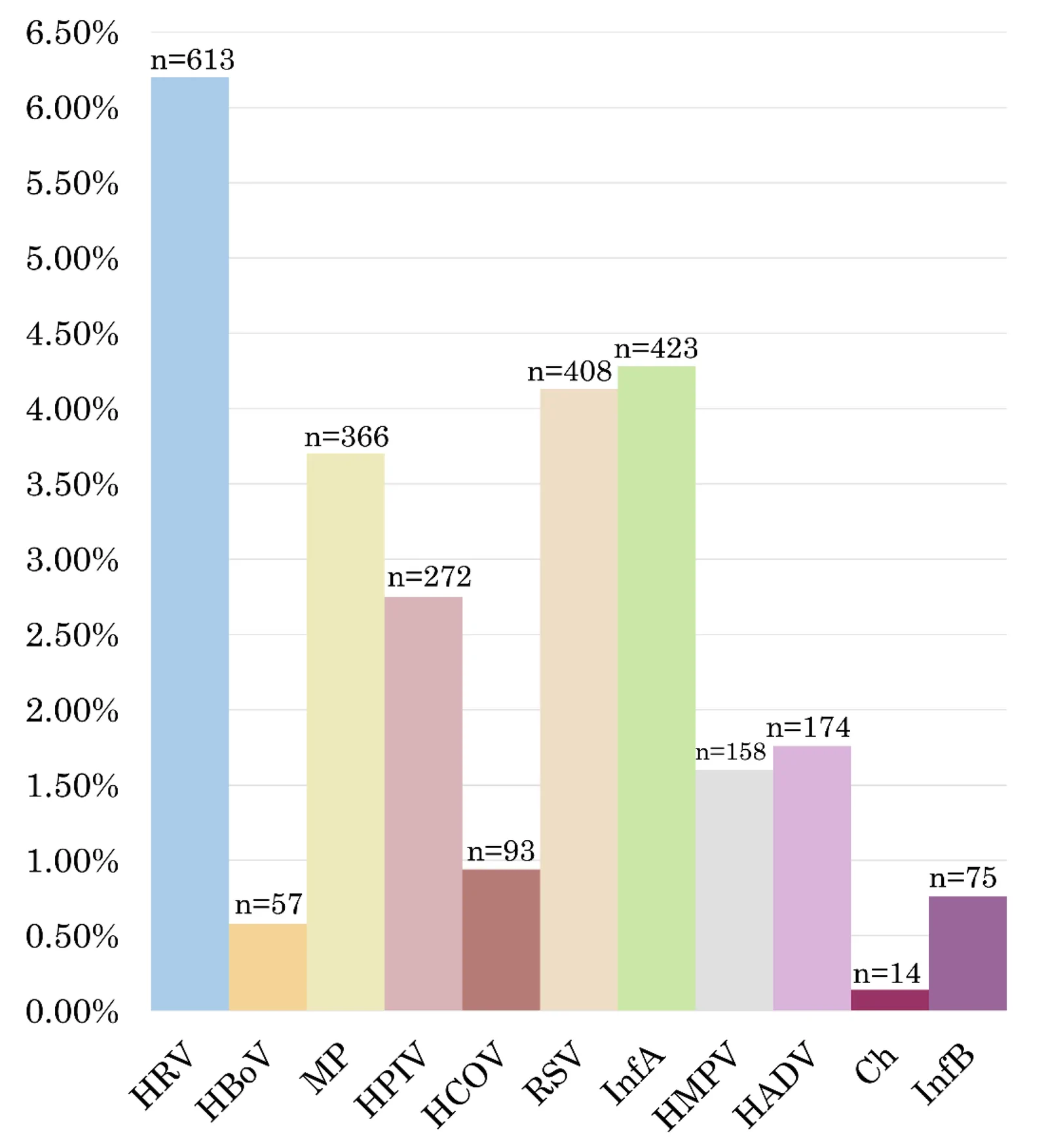
Positivity and distribution of respiratory panel targets among respiratory tract infection patients, January 2020–December 2025. InfA, influenza A virus; InfB, influenza B virus; HPIV, human parainfluenza virus; RSV, human respiratory syncytial virus; HADV, human adenoviruses; HMPV, human metapneumovirus; HRV, human rhinovirus; HBoV, human bocavirus; HCoV, human coronavirus; Ch, *Chlamydia*; MP, *Mycoplasma pneumoniae*.

A total of 9882 samples from patients with RTIs were enrolled in this study, from January 2020 to December 2025. A total of 5837 (59.07%) were from male patients, and 4045 (40.93%) were from female patients. Age ranged from 1 month to 105 years, with a median of 33 years (25th percentile–75th percentile: 4–75). Among these samples, 24.71% tested positive (n = 2442) for at least one of the target respiratory pathogens. One pathogen was detected in 2246 positive results (91.97%), while two or more targets were detected in 196 (8.03%). The detection rate of common pathogens was 26.43% (1069/4045) in females and 23.52% (1373/5837) in males. The most predominant virus was HRV, with a positivity rate of 6.20% (613 of 9882). The pathogens ranking second to fifth were InfA, RSV, MP, and HPIV, accounting for 4.28%, 4.13%, 3.70%, and 2.75%, respectively (Figure 1). Of all InfA detections (n = 423), 89 (21.04%) were showed as InfA/H1N1 and 294 (69.50%) as InfA/H3N2, and the others (n = 40) were InfA-positive with unspecified subtypes (InfA/H5N2 or H7N9).

### 3.2. Detection Percentages of Specific Pathogens from Different Ages

According to age, the patients were divided into five groups as follows: Group 1 included infants (1 month–1 year old), toddlers (1–3 years old) and pre-school children (3–6 years old), totalling 2862 cases aged from 1 month to 6 years old; Group 2 included school children and adolescents, 1178 cases aged from 7 to17; Group 3 (18–44 years old), 894 case; Group 4 (45–64 years old), 1371 case; Group 5 (≥65 years old), 3577 cases. When patients were analyzed across different age groups, positivity was higher in those aged under 18: Group 1 demonstrated the highest overall panel positivity (n = 1139, 39.79%), followed by Group 2 (n = 447, 37.95%) (Table 1).

**Figure 2:**
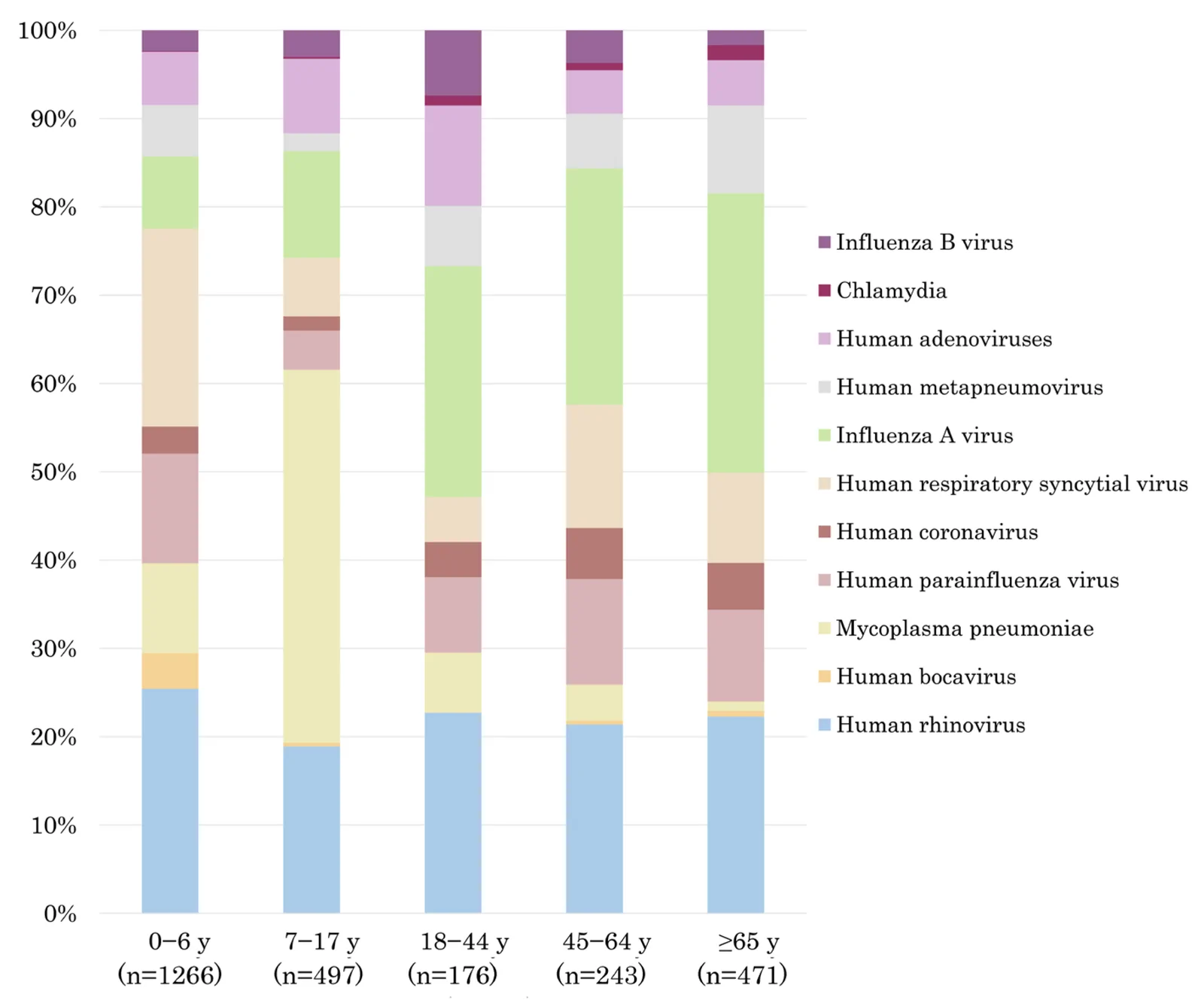
The proportions of 11 respiratory pathogens detected among different age groups. Positive detections of 11 pathogens were summarized, with the proportional contribution of each pathogen among all positive detections within each age group (number of positive detections: 0–6 years, 1266; 7–17 years, 497; 18–44 years, 176; 45–64 years, 243; and ≥65 years, 471).

Of note, due to the presence of co-detections, each individual might potentially contribute more than one count to the total number of pathogen detections. To assess whether certain pathogens were relatively more prevalent than others in specific age groups, we also calculated the relative contribution of each pathogen among all detections within each age group (number of positive detections: 0–6 years, 1266; 7–17 years, 497; 18–44 years, 176; 45–64 years, 243; and ≥65 years, 471). Positive detections were summarized with the distribution of 11 targets, and the predominant pathogens among different age groups varied (Figure 2). In patients aged 0–6 years, HRV (322/1266) was the most prevalent with a proportion of 25.43%, followed by RSV (n = 284, 22.43%) and HPIV (n = 157, 12.40%). MP accounted for the largest share (210/497, 42.25%) in those aged 7–17 years, followed by HRV (n = 94, 18.91%) and InfA (n = 60, 12.07%). In the 18–44 y group, InfA (46/176, 26.14%) was the dominant virus, followed by HRV (n = 40, 22.73%) and HADV (n = 20, 11.36%). In the 45–64 y group, InfA (65/243, 26.75%) and HRV (52/243, 21.40%) accounted for the largest proportions of positive detections, followed by RSV (n = 34, 13.99%). In patients ≥ 65 years old, InfA (149/471, 31.63%) was also the most commonly detected virus, followed by HRV (n = 105, 22.29%) and HPIV (n = 47, 9.98%). RSV and HPIV had the highest positivity in children aged 0–6 years, MP accounted for the largest share among school-aged children and adolescents, and InfA was the most prominent among adults, including elderly ≥65 years old. With the exception of InfA, HCOV and Ch, there were significant differences in pathogen detection rates among different age groups (FDR q < 0.05).

### 3.3. Detection Percentages of Specific Pathogens from Different Clinical Settings

The patients in our study were classified into the following four major types of clinical settings: Intensive care unit (ICU) (n = 1033), emergency department (including fever clinic) (n = 1249), outpatient (n = 392) and inpatient (n = 7208) settings. The overall panel positivity was highest in emergency department patients (n = 364, 29.14%), followed by outpatients (n = 112, 28.57%) (Table 2). The detection rates of InfA, InfB, MP, HADV, HMPV and HCOV were significantly different among different settings (FDR q < 0.05). Notably, the detection rates of InfA (n = 109, 8.73%), InfB (n = 19, 1.52%), HADV (n = 44, 3.52%), and HMPV (n = 37, 2.96%) were all the highest among patients from emergency department. Compared with other departments, the detection rates of RSV (n = 21, 5.36%), MP (n = 18, 4.59%), and HPIV (n = 12, 3.06%) were highest in the outpatient setting. In addition, we also found that HRV (n = 473, 6.56%) and HBoV (n = 46, 0.64%) rates were highest among inpatients.

**Figure 3:**
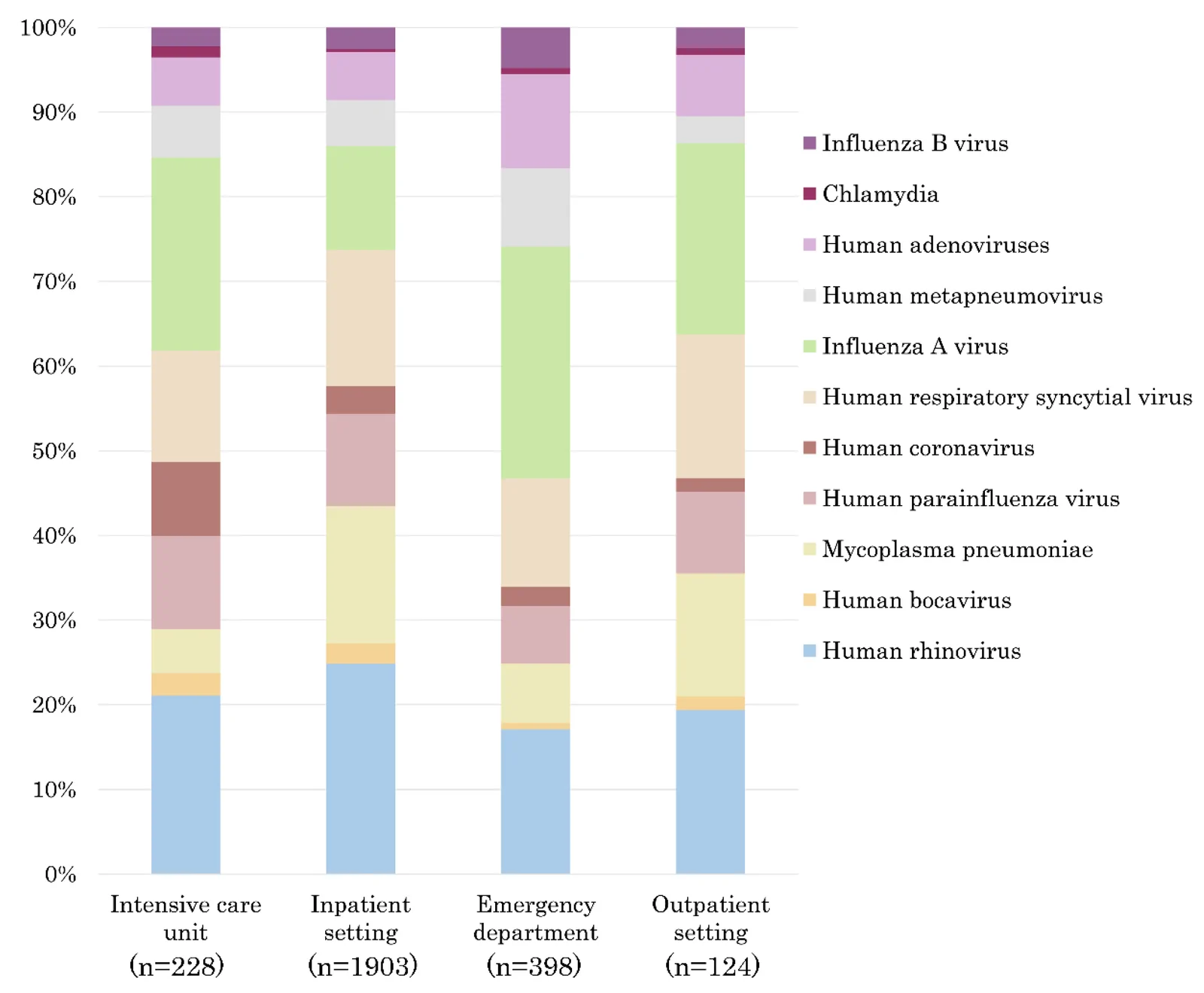
The proportions of 11 respiratory pathogens detected from major clinical settings. Positive detections of 11 pathogens were summarized, with the proportional contribution of each pathogen among all positive detections within each setting (number of positive detections: ICU, 228; inpatient setting, 1903; emergency department, 398; and outpatient setting, 124).

When pathogen contribution was calculated among all detections within each age group, the proportional composition of pathogens detected among different settings were shown in Figure 3 (number of positive detections: ICU, 228; inpatient setting, 1903; emergency department, 398; and outpatient setting, 124). A similar pattern was observed in ICU, emergency department and outpatient settings: InfA was the most predominant virus with a proportion of 22.81% (52/228), 27.39% (109/398), and 22.58% (28/124), respectively, followed by HRV and RSV. And MP was also the third most prevalent pathogen in outpatients. For inpatients, the top three pathogens were HRV, MP and RSV, accounting for 24.86% (473/1903), 16.18% (308/1903), and 16.08% (306/1903), respectively.

### 3.4. Detection Percentages of Specific Pathogens in Different Seasons

There were four groups according to the seasons: spring group (March, April and May), 2273 cases; summer group (June, July and August), 2106 cases; autumn group (September, October and November), 2688 cases; and winter group (January, February and December), 2815 cases. It was observed in Table 3 that the detection rate of common respiratory pathogens was highest in autumn (n = 893, 33.22%), followed by winter (n = 749, 26.61%). Except for Ch, there were significant differences in the detection rates among different seasons (FDR q < 0.05). Considering all examined pathogens, MP was mostly detected in autumn (n = 250, 9.30%), and InfA (n = 230, 8.17%) and RSV (n = 187, 6.64%) were detected primarily during winter. The detection rate of HPIV was highest in summer (n = 112, 5.32%), while HRV was prevalent in all seasons except winter (spring: 6.60%, summer: 5.32%, and autumn: 9.56%).

**Figure 4:**
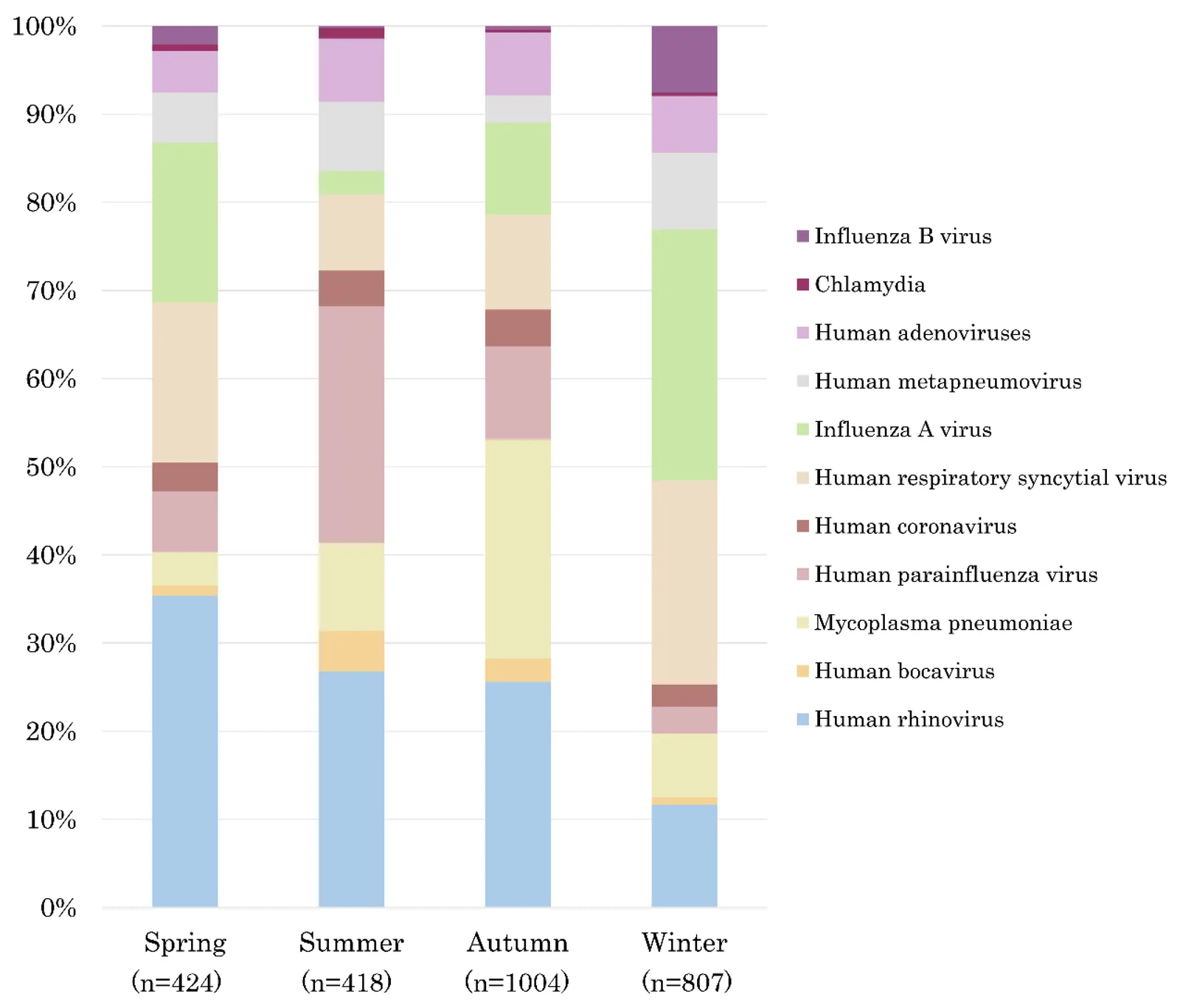
The proportions of 11 respiratory pathogens detected across seasons. Positive detections of 11 pathogens were summarized, with the proportional contribution of each pathogen among all positive detections within each season (number of positive detections: spring, 424; summer, 418; autumn, 1004; and winter, 807).

The positive results were further analyzed with the proportional distribution of the specific pathogens, which varied across seasons as shown in Figure 4 (number of positive detections: spring, 424; summer, 418; autumn, 1004; and winter, 807). In spring, HRV was the dominant virus, accounting for 35.37% (150/424), followed by InfA and RSV (n = 77, 18.16% for both). In summer, the most prevalent pathogens detected were HPIV and HRV (112/418, 26.79% for both), followed by MP (n = 42, 10.05%). In autumn, HRV accounted for the largest proportion of positive detections of 25.60% (257/1004), followed by MP (n = 250, 24.90%) and RSV (n = 108, 10.76%). In winter, the three most frequent viruses were InfA (230/807, 28.50%), RSV (n = 187, 23.17%), and HRV (n = 94, 11.64%).

### 3.5. Changes in Specific Pathogens Based on Different Years

**Figure 5:**
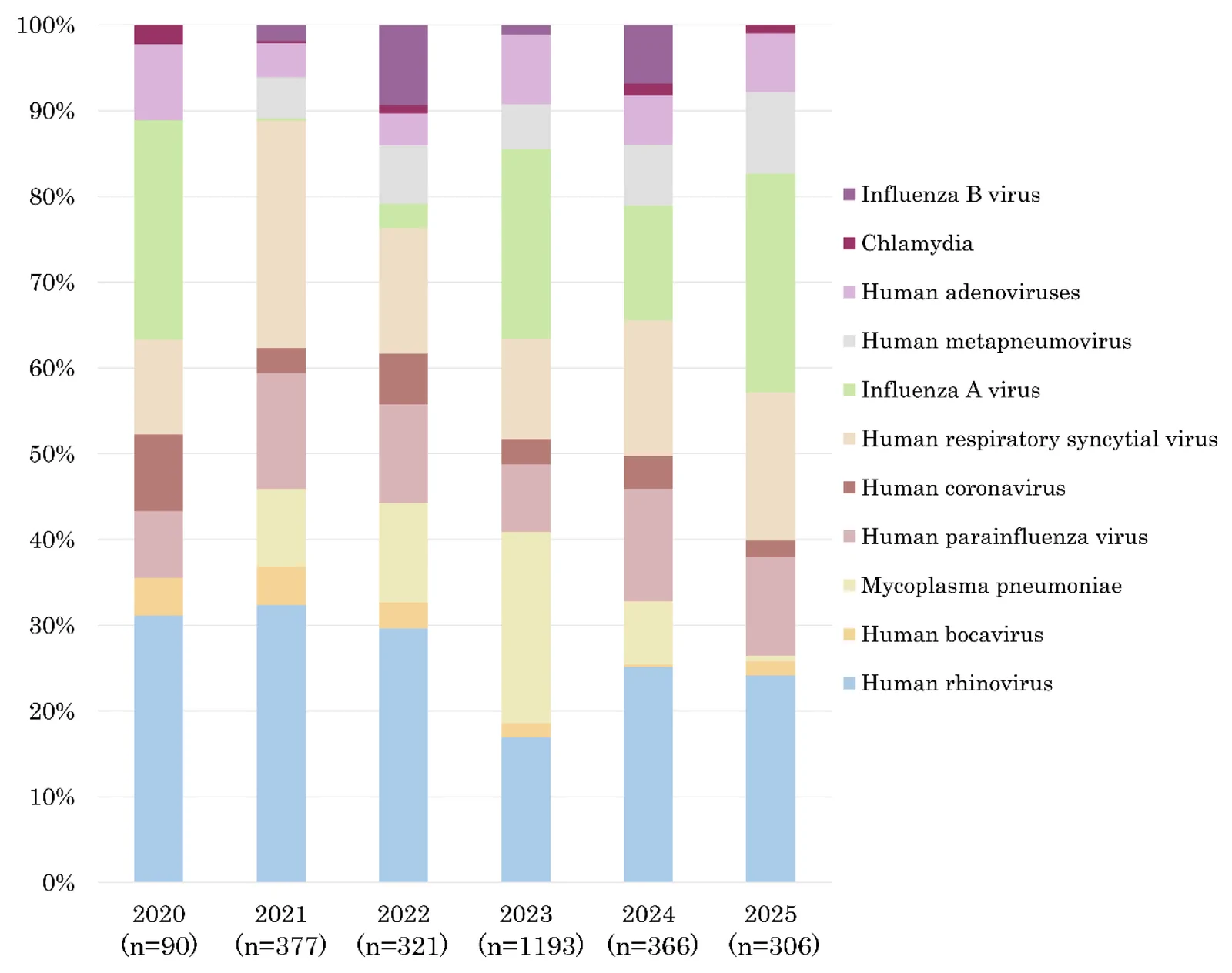
The proportions of 11 respiratory pathogens detected in different years. Positive detections of 11 pathogens were summarized, with the proportional contribution of each pathogen among all positive detections within each year (number of positive detections: 2020, 90; 2021, 377; 2022, 321; 2023, 1193; 2024, 366; and 2025, 306).

The results of detected respiratory specimens were also analyzed based on different years, including 2020 (n = 389), 2021 (n = 1013), 2022 (n = 1700), 2023 (n = 3497), 2024 (n = 1748), and 2025 (n = 1535) (Table 4 and Figure 5). The year 2021 had the highest detection rates (n = 333, 32.87%), followed by 2023 (n = 1084, 31.00%). With the exception of HCoV and HMPV, the pathogen detection rates were significantly different among different years (FDR q < 0.05). HRV was the leading virus, and RSV consistently ranked second or third throughout all years, except 2023 and 2025, whereas MP was dominant in 2023 (n = 266, 7.61%) and InfA was dominant in 2025 (n = 78, 5.08%). InfA was prevalent in 2020, 2023, 2024, and 2025, but the positivity rate was relatively low in 2021 (0.1%) and 2022 (0.53%). Monthly dynamics of testing volume and overall positivity percentages of 11 pathogens from January 2020 to December 2025 were presented in Supplementary Figure S1. Monthly total testing counts exhibited obvious temporal variation, and the distribution of positive case peaks predominantly occurred in autumn and winter. The single-month peak occurred in November 2023, with 572 specimens tested, 280 of which were positive (48.95% positivity rate), followed by December 2023 (470 total cases, 224 positive) and October 2023 (335 total cases, 154 positive).

During the COVID-19 pandemic, the Chinese government had implemented stringent NPIs until December 2022, so we also analyzed the changes during and after the pandemic. The overall positivity rate of 11 respiratory panel targets was significantly higher in 2023–2025 (n = 1723, 25.41%) than in 2020–2022 (n = 719, 23.18%). From 2020 to 2022, the respiratory pathogen spectrum was dominated by HRV (7.90%), RSV (5.06%), and HPIV (3.06%). In 2023–2025, the most frequently detected targets shifted to InfA (5.75%), HRV (5.43%) and MP (4.35%).

### 3.6. Multivariable Analysis

**Figure 6:**
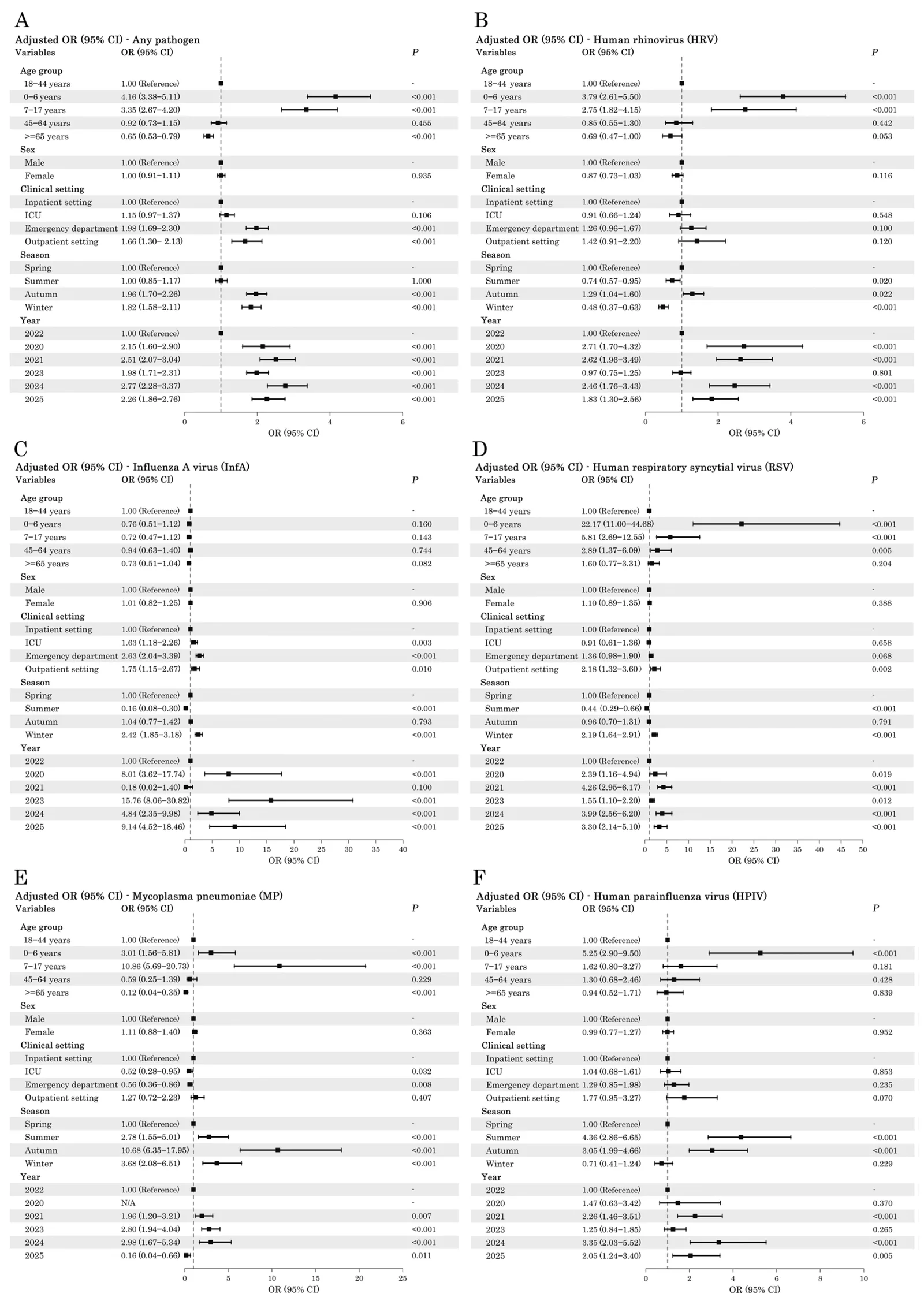
Multivariable logistic regression analysis of factors associated with detection of prevalent respiratory pathogens. (**A**) overall positivity; (**B**) HRV; (**C**) InfA; (**D**) RSV; (**E**) MP; (**F**) HPIV. Multivariable logistic regression analysis of factors associated with overall positivity (Figure 6A) and detection of five major respiratory pathogens (HRV, Figure 6B; InfA, Figure 6C; RSV, Figure 6D; MP, Figure 6E; HPIV, Figure 6F). Variables included age group, sex, season, clinical setting, and calendar year, with adults aged 18–44 years, male, spring, inpatient setting, and calendar year 2022 serving as reference categories. The following Figure shows the results of logistic regression analysis, with the *x*-axis representing the adjusted odds ratio (OR), and the *y*-axis listing the variables tested.

Multivariable logistic regression (Figure 6) analyzed associations of age group, sex, season, clinical setting, and calendar year with overall positivity (Figure 6A) and detection of five prevalent pathogens (HRV, Figure 6B; InfA, Figure 6C; RSV, Figure 6D; MP, Figure 6E; HPIV, Figure 6F). After adjustment for these variables, and using adult patients aged 18–44 years as the reference for age, children aged 0–6 years and 7–17 years had higher odds of overall detection of 11 pathogens (aOR 4.16, 95% CI: 3.38–5.11; aOR 3.35, 95% CI: 2.67–4.20), as well as HRV, RSV and MP detection; children aged 0–6 years also had higher odds of HPIV (aOR 5.25, 95% CI: 2.90–9.50); adults aged 45–64 years had higher odds of RSV (aOR 2.89, 95% CI: 1.37–6.09). Sex (reference: male) was not significantly associated with overall positivity or with detection of the five individual pathogens (all *p* > 0.05).

Regarding clinical setting (reference: inpatients), ICU was associated with higher odds of InfA (aOR 1.63, 95% CI: 1.18–2.26); emergency department was associated with higher odds of total positivity (aOR 1.98, 95% CI: 1.69–2.30) and InfA (aOR 2.63, 95% CI: 2.04–3.39); outpatient setting was also associated with higher odds of overall detection (aOR 1.66, 95% CI: 1.30–2.13), InfA (aOR 1.75, 95% CI: 1.15–2.67), and RSV (aOR 2.18, 95% CI: 1.32–3.60).

Compared with spring (reference), detection odds of MP (aOR 2.78, 95% CI: 1.55–5.01) and HPIV (aOR 4.36, 95% CI: 2.86–6.65) were higher in summer; odds of positive detection (aOR 1.96, 95% CI: 1.70–2.26), HRV (aOR 1.29, 95% CI: 1.04–1.60), MP (aOR 10.68, 95% CI: 6.35–17.95) and HPIV (aOR 3.05, 95% CI: 1.99–4.66) were higher in autumn; odds of positive detection (aOR 1.82, 95% CI: 1.58–2.11), InfA (aOR 2.42, 95% CI 1.85–3.18), RSV (aOR 2.19, 95% CI: 1.64–2.91) and MP (aOR 3.68, 95% CI: 2.08–6.51) were higher in winter.

Regarding calendar year (reference: 2022), odds of total positivity and RSV (particularly in 2021 with aOR 4.26, 95% CI: 2.95–6.17) were higher in all other years; InfA showed large differences in adjusted detection odds across calendar years (aOR 4.84–15.76); HRV was more prevalent in all years except 2023, with the highest odds in 2020 (aOR 2.71, 95% CI: 1.70–4.32); MP detection was higher in 2021, 2023 and 2024 (aOR 1.96–2.98); HPIV detection was higher in 2021 (aOR 2.26, 95% CI: 1.46–3.51), 2024 (aOR 3.35, 95% CI: 2.03–5.52) and 2025 (aOR 2.05, 95% CI: 1.24–3.40).

### 3.7. Distribution Characteristics of Co-Detection

**Figure 7:**
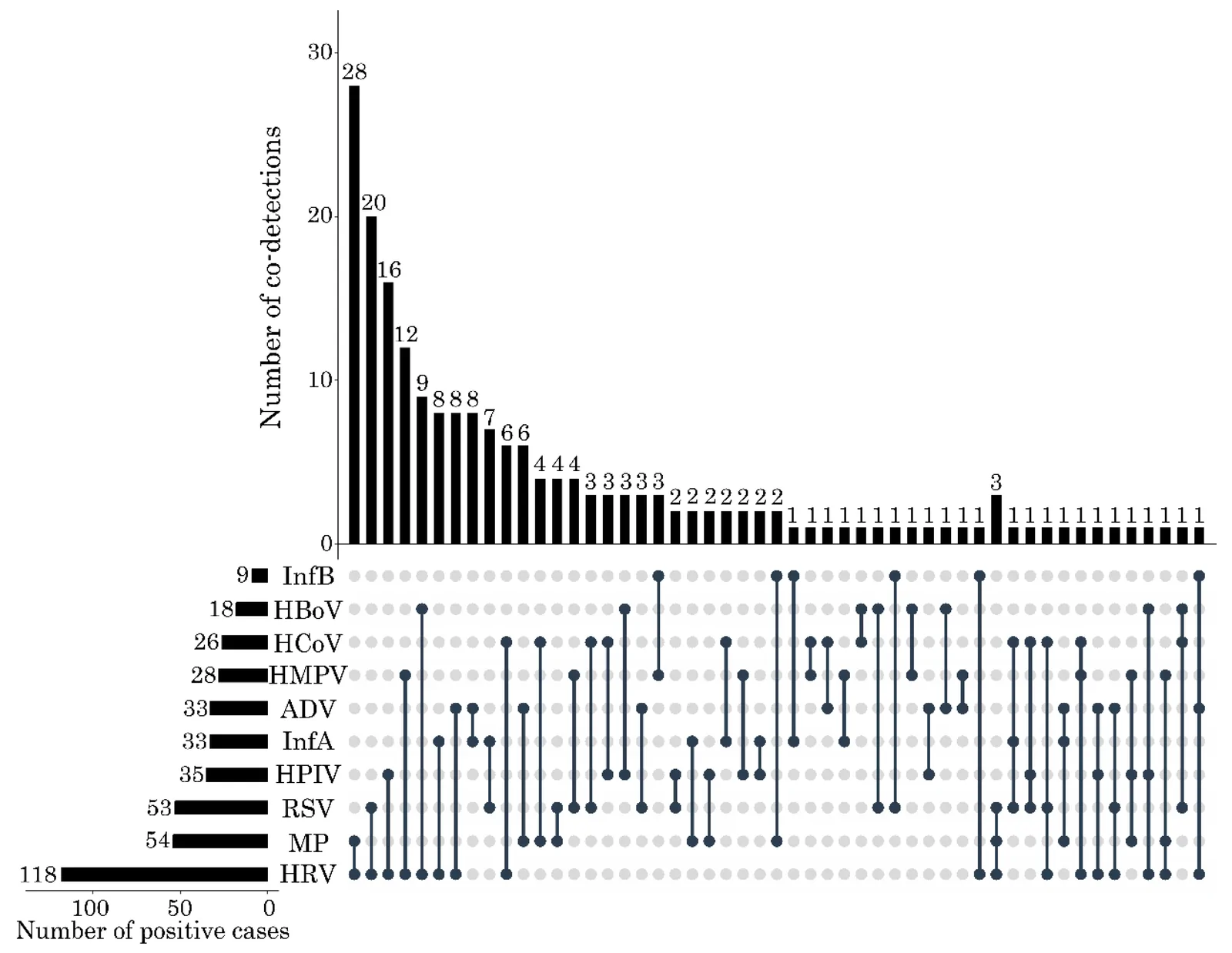
The profile of co-detections of 11 respiratory pathogens. UpSet plots of co-detection patterns included two (n = 181) or three (n = 15) targets being detected. The left horizontal bar plot shows the number of co-detection cases involving each target (Set Size). The vertical bar plot shows the number of co-detections (Intersection Size). The matrix below indicates which pathogens are present in each combination. InfA, influenza A virus; InfB, influenza B virus; HPIV, human parainfluenza virus; RSV, human respiratory syncytial virus; HADV, human adenoviruses; HMPV, human metapneumovirus; HRV, human rhinovirus; HBoV, human bocavirus; HCoV, human coronavirus; MP, *Mycoplasma pneumoniae*.

The profile of co-detection patterns among the 11 panel targets was illustrated in Figure 7. Two or more targets were detected in 196 of the 2442 positive testing cases (8.03%) and mainly (82.65%) occurred in patients under the age of 18 (1 month–6 years old: 116 cases; 7 to 17 years old: 46 cases), while the numbers of co-detection cases in other age groups were 9 cases in 18–44 years, 12 cases in 45–64 years, and 13 cases in >65 years. The majority of the co-detections involved two pathogens (n = 181, 92.35%), and three targets were detected in only 15 cases (7.65%). Among the 196 co-detection cases, the leading pathogens involved were as follows: HRV in 118 (60.20%), MP in 54 (27.55%), and RSV in 53 (27.04%). Notably, the most frequent combinations were HRV + MP (n = 28), HRV + RSV (n = 20) and HRV + HPIV (n = 16).

## 4. Discussion

This six-year, single-center hospital-based surveillance study analyzed the epidemiological and descriptive characteristics of 11 common respiratory pathogens of RTI patients at a tertiary hospital in Beijing from 2020 to 2025. The overall positivity for at least one of the 11 pathogens was 24.71%; HRV, RSV, and MP were prominent among pediatric patients, whereas InfA was the most frequently detected pathogen in adults. The positive rate of respiratory pathogens was highest in emergency department patients, with InfA being the dominant virus. MP had the highest detection rate in autumn, and InfA and RSV showed the highest rates in winter. The predominant pathogens were HRV and RSV during the COVID-19 pandemic (2020 to 2022), and InfA (2023, 2025) and MP (2023) after the pandemic. Our study demonstrated that the pathogenic positivity and distribution varied across age groups, clinical settings, seasons, and calendar years within the tested patients.

RTI is considered one of the most important health problems and ranks among the leading causes of mortality and morbidity worldwide. Respiratory pathogens are common in the general population and bring a heavy economic burden on the public health system. In our study, SARS-CoV-2 was not included in the panel, and testing results are restricted to the 11 investigated pathogens. The overall positivity for at least one of the 11 pathogens stood at 24.71%, consistent with the findings recently reported in Asia, which ranged from 17.06% to 37.2% in mainland China [14,19,20,21] and 22.03% in Taiwan, China [22] and 27.55% in Japan [23]. RTI is one of the predominant infectious diseases, primarily caused by viruses or bacteria. Viral pathogens are the most frequent cause of respiratory infections, including influenza viruses, HPIV, HRV, RSV, HADV, HMPV, coronaviruses, etc. Bacteria causing RTIs are less common than viruses but can also lead to outbreaks and sporadic cases of respiratory disease, and bacterial bronchitis or pneumonia might occur as secondary infections after viral respiratory infections [24]. We found that the major prevalent pathogens identified were HRV, InfA, RSV, and MP, which also served as the main etiological agents responsible for RTIs in other reports [20,25,26]. The present study has covered the years 2020 through 2025, a duration that spans both the implementation and withdrawal of the NPIs of the COVID-19 pandemic in China. HRV was observed as the dominant virus during this period, possibly because it was less influenced by NPIs, as it was previously reported that HRV is a non-enveloped virus that therefore might be less sensitive to disinfectants such as alcohol, thereby making it viable for an extended period of time [27,28]. Influenza is one of the common viruses related to RTIs and is regarded as the greatest threat to global health [20]. Influenza-related pneumonia is a major cause of influenza-associated hospitalizations and deaths. MP is also a vital cause of RTIs in children as well as adults that can predominantly manifest as acute respiratory infections ranging from mild to life-threatening, as reported by *China CDC Weekly* [29,30]. As we found that RSV was one of the leading pathogens, studies from several industrialized and developing countries have revealed that RSV could be associated with substantial morbidity and mortality, especially in young children [31,32,33]. Moreover, HRV and RSV are also involved in the development and/or exacerbation of asthma [1,34], a chronic inflammatory lung disease, making infections caused by these pathogens worthy of significant attention. Epidemiological investigations of RTIs can support the prevention of pathogen outbreaks to avoid wider spread.

Our study indicates that age was strongly associated with pathogen positivity. The overall detection rates were higher in underage patients (0–18 y), especially in children under six years old, compared with adults aged 18–44 y as a reference category (aOR = 4.16). The finding was similar to previous studies: although the general population is susceptible, children remain the primary source of RTIs, with younger children being more vulnerable to viral infections than older children [20,35,36], which is probably due to the immature state of children’s immune systems, predisposing them to infections. HRV accounted for the highest proportion among total enrolled subjects, and further analysis of different study groups also showed that HRV was prominent across ages, highlighting its positivity and significance as a major RTI virus. The positive rates and odds of RSV (aOR = 22.17) and HPIV (aOR = 5.25) were also substantially higher among children under 6 years old, which is fully consistent with pediatric epidemiological reports stating that they were the dominant etiology in very young children [35,37]. The affinity of RSV and HPIV for the lower respiratory tract, along with the relatively narrow airways and immature immune systems of infants and young children, often leads to more severe symptoms and hospitalization [38]. However, among patients aged 7–17 years, the detection pattern underwent a marked shift: MP and InfA were the commonly detected pathogens in addition to HRV. Notably, multivariable analysis showed that MP (aOR = 10.86) was most strongly associated with this age group and was much higher than in the others, which also aligns with reports that MP outbreaks primarily affect school-age children and adolescents [39,40]. Our study indicated that InfA accounted for the largest share of pathogen detections (approximately 5%) among all adult groups, and it also revealed that influenza viruses were mainly related to acute respiratory infections in adults (10.5% in 15–49 years) and the elderly (7.4% in 50–64 years and 6.5% in >65 years) [35]. Influenza viruses might bring a heavy burden to the healthcare system, especially in large countries like China, which has a rapidly aging population, since the positive rate of influenza was higher among adults and the elderly. It was also observed that RSV and HPIV were prevalent viruses in the 45–64-year-old age group and the ≥65-year group, respectively, which might be associated with the fact that older people are more susceptible to severe complications like pneumonia following infections [41]. The distribution of the pathogens exhibited distinct age-related differences and may help for further surveillance research and clinical prevention.

Our findings revealed that overall positivity varied among clinical settings and was highest in the emergency department (29.14%). Many RTIs are mild and self-limiting, not requiring hospitalization but requiring timely medical care, and we also found a higher positive detection in outpatients (28.57%). The emergency department exhibited the highest detection rates for InfA, InfB, HADV, and HMPV, and the highest odds of InfA compared with the inpatient setting (reference) (aOR = 2.63). Previous studies showed that influenza virus and HADV infections could cause sudden high fever and pronounced systemic symptoms, prompting patients to seek urgent medical care [21,42]. In addition, the positivity rates of MP and RSV (with a higher aOR of 2.18) were both highest in the outpatient setting, which might be partly due to the relatively stable course and the severity not requiring emergency care, leading to more frequent outpatient diagnosis and treatment. Meanwhile, HRV (6.56%) was the most prevalent pathogen among inpatients, followed by MP and RSV. A current review also demonstrated that HRV (13%) and influenza virus (7%) were the most commonly detected viral pathogens among inpatient adults [43]. However, given the cross-sectional design of this study and the lack of pre-admission specimens or baseline admission testing, it remains challenging to differentiate community-acquired infections from potential nosocomial infections. As HRV can spread through respiratory droplets, direct contact, and contaminated surfaces, possible healthcare-associated transmission should also be considered. Further prospective investigations with admission-time sampling and ward-level epidemiological investigation are needed to evaluate the contribution of nosocomial HRV transmission. Moreover, we also observed a similar pattern in ICU, emergency department, and outpatient settings (except the inpatient setting), as InfA was the most predominant virus, followed by HRV and RSV. When influenza virus becomes a global concern, laboratory testing may be useful for respiratory infection patients with suspected influenza and for those whose treatment decisions would be improved upon a confirmed diagnosis [42].

This study further found that the detection rates of 11 respiratory pathogens exhibited clear seasonal variation, with the highest rate and detection odds (reference: spring) in autumn (33.22%, aOR = 1.96), followed by winter (26.61%, aOR = 1.82), which is consistent with current investigations reporting that some respiratory viruses had seasonal trends with annual peaks in the fall and winter [26,44]. However, this was different from a previous study which showed that spring had the highest positive rate in Shenzhen, a city located in southern China with a typical subtropical monsoon climate quite different from that in Beijing [37]. The above findings indicated that seasonal characteristics of respiratory pathogens might be related to a region’s climate and demographic factors. Furthermore, the positivity percentage of different pathogens varies across four seasons. Previous investigations have revealed that MP outbreaks primarily occur in autumn, mainly affecting school-age minors [39,40]. Similarly, we observed that MP demonstrated its highest detection rate and significant association with autumn (aOR = 10.68), which was potentially related to increased school attendance and gatherings among school-aged populations following the start of the fall semester. InfA and RSV showed the highest positive rates in winter, exhibiting typical seasonal patterns, as current multivariable analysis and the previous findings noted [44,45]. In addition, HRV was prevalent in spring and autumn, whereas HPIV predominated in summer—an epidemiological feature consistently observed in earlier studies [46]. These observed seasonal patterns may help emphasize the importance of the national surveillance system and help to formulate questions for broader surveillance studies, particularly for high-risk groups such as infants, young children, and elderly individuals. A multi-province study of hospitalized acute lower respiratory infections from 81 sentinel hospitals in 22 provinces showed that RSV and influenza virus had annual peaks in northern China but biannual peaks in southern China, while HPIV, HBoV, and HMPV showed pathogen-specific seasonal patterns; meteorological factors such as temperature, atmospheric pressure, vapor pressure, rainfall, humidity, sunlight hours, and wind speed contributed differently by pathogen and region [47]. In particular, MP and HBoV positivity may represent carriage or persistent detection rather than the cause of the presenting illness. These findings reinforce the value of integrated post-COVID respiratory surveillance frameworks capable of monitoring SARS-CoV-2, influenza, RSV, other respiratory viruses, and atypical bacterial pathogens within shared laboratory platforms [48].

Since the end of 2019, severe acute respiratory syndrome coronavirus 2 has spread as a new strain of coronavirus disease (COVID-19) and progressed as a global pandemic. The Chinese government implemented stringent NPIs until December 2022, when it announced that COVID-19 patients did not need to be quarantined; within the following month, a significant proportion of people were infected with SARS-CoV-2. Our study showed that the total pathogen detection rate was relatively lower during 2020–2022, likely due to the NPIs implemented in response to COVID-19, as reported [26]. COVID-19 interventions and behavioral changes markedly reduced the activity of multiple viruses in China, reflecting that urbanization, population density, human contact, mobility, and migration may influence respiratory illness dynamics [49]. *China CDC Weekly* also noted the profound reshaping of pathogen ecology after the COVID-19 pandemic [50]. In this single-center descriptive study, the dominant pathogens were HRV and RSV during the COVID-19 pandemic (2020 to 2022). Many studies have shown that NPIs associated with reducing the spread of COVID-19 might also substantially decrease the transmission of influenza and delay its return in the community [25,51]. In contrast, as a non-enveloped virus, HRV maintained relative persistence with its structurally stable nature and was less sensitive to disinfectants [27,28]. After the COVID-19 pandemic, our study indicated that the positivity of InfA (aOR = 15.76 in 2023 and aOR = 9.14 in 2025, compared with 2022 as reference category) and MP was increased (aOR = 2.80 in 2023), which aligned with a previous report from *China CDC Weekly* [29]. Notably, MP reached its peak in autumn and spread widely among the school-age population, forming a distinct MP epidemic in 2023, which might indicate that the respiratory pathogens began to resume their original transmission characteristics, triggering widespread infections among the susceptible population [26,29]. These findings suggest that the outbreaks of multiple respiratory diseases, including but not limited to InfA and MP, have highlighted the complexity of public health crisis management [29]. However, apart from the lifting of NPIs, the shift in the pathogen spectrum or the observed differences between the 2020–2022 and 2023–2025 periods could also result from changes in healthcare-seeking behavior, testing indications, composition of patients, pathogen circulation, and vaccination coverage. In addition to changes in pathogen circulation in the general population, temporal differences in positivity might also reflect variations in testing indications, patient selection, or composition.

The present study found a co-detection rate of 8.03% (196 of 2442) in positive cases, lower than that in previous research, accounting for 12.1%, with the majority being double co-infections [20]. Notably, we observed that 82.65% of the co-detections occurred in patients under 18 y, which might be related to frequent exposure and high background positivity of several pediatric respiratory pathogens [20,33,38]. The immaturity of children’s immune systems may also be associated with their heightened susceptibility to multiple respiratory pathogens [38]. It should be noted that in our study, HRV was involved in 60.20% of co-detections, possibly reflecting the high epidemiological positivity of HRV [20,25]. MP and RSV are the second and third most commonly detected pathogens in co-detections, respectively, which is consistent with their characteristics as prevalent pathogens in children’s respiratory infections [29,33]. The most common co-detection combinations in this study are HRV + MP, HRV + RSV, and HRV + HPIV. Because SARS-CoV-2 and other targets were not assessed, the reported combinations do not provide a complete account of all organisms potentially present. Other studies have reported co-detection in pediatric respiratory testing [20,30,38], but these findings do not establish that all detected organisms caused active disease or interacted biologically. Since nucleic acid detection/co-detection may represent an active infection, asymptomatic carriage, or residual viral DNA/RNA fragments, not establishing that all detected pathogens caused clinically active infection or interacted with one another, it is necessary to continuously monitor viral epidemiological trends and conduct additional investigation. However, future respiratory disease control should also shift from single-pathogen surveillance to a comprehensive multi-pathogen monitoring system, especially in young children [29].

However, our research has certain limitations, partly because it was only focused on RTI patients from one tertiary hospital in Beijing, which might have led to enrollment bias considering the geographic location. Changes in testing indications, healthcare access, healthcare-seeking behavior, and patient composition may have influenced the observed results. Adjustment for recorded covariates cannot remove these unmeasured influences, and their magnitude and direction cannot be determined from the available data. Our regression models did not account for within-patient correlation, which might have led to underestimated standard errors or narrow CIs when episodes from the same patient are correlated. Some clinical information, such as comorbidities, vaccination history, disease severity, and antibiotic and antiviral treatment, was not included in the original analytical database and limited comparisons across different settings. The molecular testing panel was restricted to 11 pathogens and did not assess SARS-CoV-2 or other potentially relevant bacterial, fungal, and viral pathogens; thus, total pathogen positivity and co-detection rates were presumably underestimated, and included targets might have been detected alongside untested pathogens. We therefore could not assess SARS-CoV-2 co-detection or its contribution to the observed patterns. Molecular detection alone cannot distinguish active etiological infection from carriage or persistent nucleic acid detection, particularly for MP and HBoV. Our study is also limited by a preliminary and retrospective cross-sectional design. Therefore, a multicenter, longitudinal, prospective investigation with standardized testing criteria and broader pathogen coverage is required to address the etiological and population-level questions that these data cannot resolve.

## 5. Conclusions

This six-year hospital-based surveillance study in a single center identified marked age, clinical-setting, seasonal, and annual variation in the positivity of respiratory pathogens among RTIs. HRV was the most frequently detected virus overall. HRV, RSV, and MP were prominent among pediatric patients, while InfA accounted for the largest share of pathogen detections in adult groups. The positivity percentage of the 11 observed respiratory pathogens was highest in the emergency department, with InfA as the dominant virus. MP had the highest positivity percentage in autumn, and InfA was the most frequent virus in winter. The dominant pathogens were HRV and RSV during 2020–2022, and they shifted to InfA, MP and HRV during 2023–2025. The co-detections mainly occurred in patients under the age of 18, and the most common patterns were HRV + MP, HRV + RSV, and HRV + HPIV. Multivariable analysis showed that age, clinical setting, season, and calendar year were associated with overall and prevalent pathogen detections. Thus, differentiated prevention should be developed for different patients, particularly for high-risk groups such as infants, young children, and elderly individuals.

## Figures and Tables

**Table 1:** Detection percentages of respiratory pathogens based on different ages.

Age (Years)	Positive Episodes (N, %)	HRV (n, %)	HBoV (n, %)	MP (n, %)	HPIV (n, %)	HCoV (n, %)	RSV (n, %)	InfA (n, %)	HMPV (n, %)	HADV (n, %)	Ch (n, %)	InfB (n, %)
0–6	1139 (39.80)	322 (11.25)	51 (1.78)	129 (4.51)	157 (5.49)	39 (1.36)	284 (9.92)	103 (3.60)	74 (2.59)	76 (2.66)	1 (0.03)	30 (1.05)
7–17	447 (37.95)	94 (7.98)	2 (0.17)	210 (17.83)	22 (1.87)	8 (0.68)	33 (2.80)	60 (5.09)	10 (0.85)	42 (3.57)	1 (0.08)	15 (1.27)
18–44	167 (18.68)	40 (4.47)	0 (0.00)	12 (1.34)	15 (1.68)	7 (0.78)	9 (1.01)	46 (5.15)	12 (1.34)	20 (2.24)	2 (0.22)	13 (1.45)
45–64	230 (16.78)	52 (3.79)	1 (0.07)	10 (0.73)	29 (2.12)	14 (1.02)	34 (2.48)	65 (4.74)	15 (1.09)	12 (0.88)	2 (0.15)	9 (0.66)
≥65	459 (12.83)	105 (2.94)	3 (0.08)	5 (0.14)	49 (1.37)	25 (0.70)	48 (1.34)	149 (4.17)	47 (1.31)	24 (0.67)	8 (0.22)	8 (0.22)
unadjusted *p*	<0.001	<0.001	<0.001	<0.001	<0.001	0.063	<0.001	0.107	<0.001	<0.001	0.320	<0.001
q (FDR)	<0.001	<0.001	<0.001	<0.001	<0.001	0.081	<0.001	0.128	<0.001	<0.001	0.345	<0.001
Cramér’s V (95% CI)	0.284 (0.265–0.303)	0.148 (0.130–0.168)	0.102 (0.084–0.119)	0.291 (0.265–0.319)	0.108 (0.088–0.131)	0.030 (0.017–0.053)	0.188 (0.167–0.209)	0.028 (0.016–0.052)	0.052 (0.034–0.075)	0.083 (0.067–0.104)	0.022 (0.013–0.043)	0.052 (0.040–0.073)

Positive episodes (%) refer to the number of positive counts among the total number of cases tested in each group (0–6 y: 2862 cases; 7–17 y: 1178 cases; 18–44 y: 894 cases; 45–64 y: 1371 cases; ≥65 y: 3577 cases). Due to the presence of co-detections, each individual might potentially contribute more than one count to the number of pathogen detections. The differences among the study groups were examined, with *p*-values adjusted for multiple comparisons using the Benjamini–Hochberg false discovery rate (FDR; q = 0.05).

**Table 2:** Detection percentages of respiratory pathogens from different clinical settings.

Clinical Settings	Positive Episodes (N, %)	HRV (n, %)	HBoV (n, %)	MP (n, %)	HPIV (n, %)	HCoV (n, %)	RSV (n, %)	InfA (n, %)	HMPV (n, %)	HADV (n, %)	Ch (n, %)	InfB (n, %)
ICU	217 (21.01)	48 (4.65)	6 (0.58)	12 (1.16)	25 (2.42)	20 (1.94)	30 (2.90)	52 (5.03)	14 (1.36)	13 (1.26)	3 (0.29)	5 (0.48)
Inpatient setting	1749 (24.26)	473 (6.56)	46 (0.64)	308 (4.27)	208 (2.89)	62 (0.86)	306 (4.25)	234 (3.25)	103 (1.43)	108 (1.50)	7 (0.10)	48 (0.67)
Emergency department	364 (29.14)	68 (5.44)	3 (0.24)	28 (2.24)	27 (2.16)	9 (0.72)	51 (4.08)	109 (8.73)	37 (2.96)	44 (3.52)	3 (0.24)	19 (1.52)
Outpatient setting	112 (28.57)	24 (6.12)	2 (0.51)	18 (4.59)	12 (3.06)	2 (0.51)	21 (5.36)	28 (7.14)	4 (1.02)	9 (2.30)	1 (0.26)	3 (0.77)
unadjusted *p*	<0.001	0.068	0.396	<0.001	0.445	0.005	0.129	<0.001	<0.001	<0.001	0.279	0.009
q (FDR)	<0.001	0.085	0.419	<0.001	0.462	0.007	0.148	<0.001	0.001	<0.001	0.307	0.013
Cramér’s V (95% CI)	0.050 (0.032–0.072)	0.027 (0.013–0.047)	0.017 (0.009–0.035)	0.058 (0.046–0.074)	0.016 (0.007–0.038)	0.036 (0.016–0.064)	0.024 (0.012–0.046)	0.095 (0.069–0.121)	0.042 (0.021–0.070)	0.053 (0.032–0.081)	0.020 (0.009–0.051)	0.034 (0.014–0.063)

Positive episodes (%) refer to the number of positive counts among the total number of cases tested in each group (ICU: 1033 cases; inpatient setting: 7208 cases; emergency department: 1249 cases; and outpatient setting: 392 cases). Due to the presence of co-detections, each individual might potentially contribute more than one count to the number of pathogen detections. The differences among the study groups were examined, with *p*-values adjusted for multiple comparisons using the Benjamini–Hochberg false discovery rate (FDR; q = 0.05).

**Table 3:** Detection percentages of respiratory pathogens in different seasons.

Season	Positive Episodes (N, %)	HRV (n, %)	HBoV (n, %)	MP (n, %)	HPIV (n, %)	HCoV (n, %)	RSV (n, %)	InfA (n, %)	HMPV (n, %)	HADV (n, %)	Ch (n, %)	InfB (n, %)
Spring	410 (18.04)	150 (6.60)	5 (0.22)	16 (0.70)	29 (1.28)	14 (0.62)	77 (3.39)	77 (3.39)	24 (1.06)	20 (0.88)	3 (0.13)	9 (0.40)
Summer	390 (18.52)	112 (5.32)	19 (0.90)	42 (1.99)	112 (5.32)	17 (0.81)	36 (1.71)	11 (0.52)	33 (1.57)	30 (1.42)	5 (0.24)	1 (0.05)
Autumn	893 (33.22)	257 (9.56)	26 (0.97)	250 (9.30)	106 (3.94)	42 (1.56)	108 (4.02)	105 (3.91)	31 (1.15)	72 (2.68)	3 (0.11)	4 (0.15)
Winter	749 (26.61)	94 (3.34)	7 (0.25)	58 (2.06)	25 (0.89)	20 (0.71)	187 (6.64)	230 (8.17)	70 (2.49)	52 (1.85)	3 (0.11)	61 (2.17)
unadjusted *p*	<0.001	<0.001	<0.001	<0.001	<0.001	0.001	<0.001	<0.001	<0.001	<0.001	0.616	<0.001
q (FDR)	<0.001	<0.001	<0.001	<0.001	<0.001	0.002	<0.001	<0.001	0.001	<0.001	0.616	<0.001
Cramér’s V (95% CI)	0.145 (0.126–0.165)	0.098 (0.079–0.118)	0.047 (0.031–0.065)	0.183 (0.163–0.206)	0.111 (0.093–0.129)	0.040 (0.021–0.063)	0.090 (0.072–0.110)	0.136 (0.119–0.155)	0.047 (0.028–0.069)	0.050 (0.033–0.073)	0.014 (0.006–0.039)	0.103 (0.084–0.123)

Positive episodes (%) refer to the number of positive counts among the total number of cases tested in each group (spring: 2273 cases, summer: 2106 cases, autumn: 2688 cases, and winter: 2815 cases). Due to the presence of co-detections, each individual might potentially contribute more than one count to the number of pathogen detections. The differences among the study groups were examined, with *p*-values adjusted for multiple comparisons using the Benjamini–Hochberg false discovery rate (FDR; q = 0.05).

**Table 4:** Changes in respiratory pathogens based on different years.

Year	Positive Episodes (N, %)	HRV (n, %)	HBoV (n, %)	MP (n, %)	HPIV (n, %)	HCoV (n, %)	RSV (n, %)	InfA (n, %)	HMPV (n, %)	HADV (n, %)	Ch (n, %)	InfB (n, %)
2020	80 (20.57)	28 (7.2)	4 (1.03)	0 (0)	7 (1.8)	8 (2.06)	10 (2.57)	23 (5.91)	0 (0)	8 (2.06)	2 (0.51)	0 (0)
2021	333 (32.87)	122 (12.04)	17 (1.68)	34 (3.36)	51 (5.03)	11 (1.09)	100 (9.87)	1 (0.1)	18 (1.78)	15 (1.48)	1 (0.1)	7 (0.69)
2022	306 (18.00)	95 (5.59)	10 (0.59)	37 (2.18)	37 (2.18)	19 (1.12)	47 (2.76)	9 (0.53)	22 (1.29)	12 (0.71)	3 (0.18)	30 (1.76)
2023	1084(31.00)	202 (5.78)	20 (0.57)	266 (7.61)	94 (2.69)	35 (1)	140 (4)	263 (7.52)	63 (1.8)	97 (2.77)	0 (0)	13 (0.37)
2024	347 (19.85)	92 (5.26)	1 (0.06)	27 (1.54)	48 (2.75)	14 (0.8)	58 (3.32)	49 (2.8)	26 (1.49)	21 (1.2)	5 (0.29)	25 (1.43)
2025	292 (19.02)	74 (4.82)	5 (0.33)	2 (0.13)	35 (2.28)	6 (0.39)	53 (3.45)	78 (5.08)	29 (1.89)	21 (1.37)	3 (0.20)	0 (0.00)
unadjusted *p*	<0.001	<0.001	<0.001	<0.001	<0.001	0.043	<0.001	<0.001	0.094	<0.001	0.036	<0.001
q (FDR)	<0.001	<0.001	<0.001	<0.001	<0.001	0.057	<0.001	<0.001	0.115	<0.001	0.049	<0.001
Cramér’s V (95% CI)	0.144 (0.123–0.162)	0.084 (0.063–0.112)	0.058 (0.038–0.087)	0.160 (0.142–0.178)	0.050 (0.031–0.078)	0.034 (0.022–0.063)	0.100 (0.077–0.130)	0.144 (0.132–0.160)	0.031 (0.028–0.050)	0.061 (0.045–0.083)	0.035 (0.028–0.067)	0.075 (0.060–0.094)
2020–2022	719 (23.18)	245 (7.90)	31 (1.00)	71 (2.29)	95 (3.06)	38 (1.23)	157 (5.06)	33 (1.06)	40 (1.29)	35 (1.13)	6 (0.19)	37 (1.19)
2023–2025	1723 (25.41)	368 (5.43)	26 (0.38)	295 (4.35)	177 (2.61)	55 (0.81)	251 (3.70)	390 (5.75)	118 (1.74)	139 (2.05)	8 (0.12)	38 (0.56)
unadjusted *p*	0.017	<0.001	0.001	<0.001	0.227	0.062	0.002	<0.001	0.116	0.002	0.524	0.001
q (FDR)	0.017	<0.001	0.001	<0.001	0.255	0.081	0.003	<0.001	0.136	0.003	0.534	0.002
Odds ratio (95% CI)	0.89 (0.80–0.9)	1.49 (1.26–1.77)	2.62 (1.55–4.42)	0.51 (0.40–0.67)	1.18 (0.92–1.52)	1.52 (1.00–2.30)	1.39 (1.13–1.70)	0.18 (0.12–0.25)	0.74 (0.51–1.06)	0.55 (0.38–0.79)	1.64 (0.57–4.73)	2.14 (1.36–3.37)

Positive episodes (%) refer to the number of positive counts among the total number of cases tested in each group (2020: 389 cases; 2021: 1013 cases; 2022: 1700 cases; 2023: 3497 cases; 2024: 1748 cases; 2025: 1535 cases). Due to the presence of co-detections, each individual might potentially contribute more than one count to the number of pathogen detections. The differences among the study groups were examined, with *p*-values adjusted for multiple comparisons using the Benjamini–Hochberg false discovery rate (FDR; q = 0.05).

## Data Availability

The data presented in this study are available on reasonable request from the corresponding author. The data are not publicly available due to privacy and ethical restrictions.

## Supplementary Materials

The following supporting information can be downloaded at: https://www.mdpi.com/article/10.3390/jcm15218742/s1; Figure S1. Monthly distribution and positivity percentage of respiratory panel testing results, January 2020–December 2025.
